# Immunogenicity of SARS-CoV-2 mRNA intramuscular vaccination in patients with muscular disorders

**DOI:** 10.3389/fimmu.2023.1103196

**Published:** 2023-02-07

**Authors:** Ryousuke Kasai, Michinori Funato, Kanako Maruta, Kunihiko Yasuda, Hiroshi Minatsu, Junji Ito, Kazuhiro Takahashi

**Affiliations:** ^1^ Department of Pediatrics, National Hospital Organization Nagara Medical Center, Gifu, Japan; ^2^ Department of Pediatric Neurology, National Hospital Organization Nagara Medical Center, Gifu, Japan; ^3^ Department of Pediatric Surgery, National Hospital Organization Nagara Medical Center, Gifu, Japan; ^4^ Department of Clinical Examination, National Hospital Organization Nagara Medical Center, Gifu, Japan

**Keywords:** COVID-19, muscular disorder, severe motor and intellectual disabilities, immunization, antibody responses

## Abstract

**Backgrounds:**

Little clinical data is available on severe acute respiratory syndrome coronavirus 2 (SARS-CoV-2) in patients with muscular disorders (MDs). The immunogenicity of SARS-CoV-2 vaccines against MDs, in particular, remains unknown. Thus, this study aimed to confirm the immunogenicity and safety of the SARS-CoV-2 vaccine against MDs.

**Methods:**

All participants were vaccinated with two doses of mRNA vaccines (BNT162b2, Pfizer-BioNTech). The serum samples were collected from each patient on the day of second dose of vaccination, and then, consecutively, after one month, three months, and six months. Anti-SARS-CoV-2 IgG levels were determined using the Abbott SARS-CoV-2 IgG II Quant assay.

**Results:**

We evaluated 75 individuals, including 42 patients with MDs and 33 patients with non-muscular disorders (non-MDs). Non-MD patients primarily include those with severe motor and intellectual disabilities. The median age of the patients was 32 years (range 12–64 years). After one and three months following the second immunization, patients with MDs had lower antibody responses. Furthermore, three months following the second immunization, the proportion of high responders among patients with MDs decreased significantly compared to that among patients without MDs (*p*-value of less than 0.01). No serious adverse events were observed in patients with or without MDs.

**Conclusion:**

Intensity and latency of antibody response were suppressed in patients with MDs. Although MDs may be a key contributor in predicting the antibody response to SARS-CoV-2 vaccination, SARS-CoV-2 immunization in MDs needs extensive research.

## Introduction

The novel coronavirus disease-19 (COVID-19), caused by the severe acute respiratory syndrome coronavirus 2 (SARS-CoV-2), is on the verge of being endemic globally. The mRNA vaccines BNT162b2 (Pfizer-BioNTech) and mRNA-1273 (Moderna-NIAID) have been significant in protecting against the severe COVID-19 (1, 2). Patients with muscular disorders (MDs) are likely at a higher risk of COVID-19, notably advanced Duchenne muscular dystrophy (DMD), because most of them have chronic respiratory tract and heart diseases accompanied with or without chronic renal illnesses (3, 4). Despite recent clinical case reports describing complete recovery of patients with DMD without complications from SARS-CoV-2 infection, the prognosis remains unknown (5, 6). Additionally, the vaccine’s efficacy remains undetermined because it has not been clearly described yet. Therefore, further investigation into the effects of SARS-CoV-2 infection and vaccination in patients with MDs is required.

MDs are often associated with progressive or degenerative muscular weakness, so evaluating whether SARS-CoV-2 mRNA intramuscular immunization affects patients with MDs is crucial. Demeonbreun et al. and Iwayama et al. reported that intramuscular mRNA immunization resulted in a strong IgG antibody response in patients with advanced MDs (7, 8). However, there are limited reports on the effect of SARS-CoV-2 vaccines on MDs, in particular for long periods.

In this work, anti-SARS-CoV-2 IgG antibodies were measured in patients with MDs *vs.* patients with non-muscular disorders (non-MDs), primarily including those with severe motor and intellectual disabilities (SMIDs) using the Abbott Architect SARS-CoV-2 IgG immunoassay. Moreover, we discussed the findings on the effect of SARS-CoV-2 vaccines on MDs.

## Subjects and methods

### Ethical compliance

This study was approved by the Nagara Medical Center’s ethics committee (Approval number/ID 2021-14 and 2021-20). Before the study began, written informed consent was obtained from each patient. When patients had learning disabilities, written informed consent was obtained from each parent.

### Subjects

This study included a total of 83 patients who had been outpatients or inpatients at the Nagara Medical Center. They were 12 years of age or older and hoped to be vaccinated at the Nagara Medical Center. All inpatients were admitted for long-term care or treatment. The patients had a history of the following diseases or disorders: DMD (one patient treated with chronic glucocorticoid steroids), female carriers of DMD (DMD-carrier), Myotonic dystrophy type 1 (DM1), Fukuyama congenital muscular dystrophy (FCMD), Myotubular myopathy (MTM), Distal myopathy with rimmed vacuoles (DMRV), Limb-girdle muscular dystrophy (LGMD), Spinal muscular atrophy (SMA), Cerebral palsy caused by Hypoxic-ischemic encephalopathy, Asphyxia, Kernicterus or unknown, sequela of brain infarction or Reye’s syndrome, Angelman syndrome, Rett syndrome, Miller-Dieker syndrome, Nodular sclerosis, Spinocerebellar degeneration, Chromosome anomaly, and malformation syndrome. All were vaccinated with two doses of BNT162b2 vaccines. according to the package insert, which was approved by the Ministry of Health, Labour and Welfare of Japan (9). In addition, all had no clinical history of confirmed SARS-CoV-2 infection before the commencement of the study. Patients with severe muscular atrophy were injected into the deltoid muscle under muscular echographic supervision. Four aliquots of each 3 mL blood sample were collected into tubes containing a clot activator and serum gel separator from each patient: on the day of the second vaccination (i.e., day 0), 21–35 days (i.e., one month after the second vaccination), 77–91 days (i.e., 3 months after the second vaccination), and 161–175 days (i.e., 6 months after the second vaccination). To obtain serum samples, the blood samples were centrifuged, and the serum was frozen at -20°C. The patients who were unable to undergo blood sampling within the allotted time frame were eliminated from the study.

### Evaluation

Using a chemiluminescent microparticle immunoassay, i.e., an Abbott Architect SARS-CoV-2 IgG Quant II (Abbott, Sligo, Ireland), we examined the levels of anti-SARS-CoV-2 IgG antibodies according to the manufacturer’s instructions (10). This technique detects IgG antibodies directed to the receptor-binding domain of the S1 subunit SARS-CoV-2 spike protein. Briefly, the serum was incubated with SARS-CoV-2 antigen coated paramagnetic microparticles, to form immune complexes. After washing, acridinium-labeled anti-human IgG was added and incubated with the sample. Pre-trigger and trigger solutions were added following a wash cycle. The chemiluminescent reaction was measured as relative light units. The analytical measurement range as defined by the manufacturer is 3–5,680 BAU/mL, and the cut-off is ≥7.1 BAU/mL. The immunogenicity is defined as: Negative (<7.1 BAU/mL), Low positive (7.1–119.2 BAU/mL), Moderate positive (119.3–567.9 BAU/mL), and High positive (>568 BAU/mL) (11).

Systemic and local adverse effects within seven days of receiving the second dose of the BNT162b2 vaccine were retrospectively recorded. As a systemic adverse impact, fever was detected, whereas local swelling, pain, redness, and warmth at or near the injection site were reported as local adverse effects. These symptoms were reported by the patient’s mother for outpatients and by hospital staff for inpatients due to the patient’s intellectual disability.

### Statistical analysis

The statistical significance of the data regarding the levels of anti-SARS-CoV-2 IgG antibodies (MDs *vs*. non-MDs, female *vs*. male, outpatients *vs*. inpatients, under the age of 30 *vs*. over the age of 31, mechanical ventilation *vs*. none, tube feeding *vs*. oral feeding) was determined using the paired t-test. A chi-square test of independence was conducted by comparing the incidence of favorable immune responses after three months following the second immunization (MDs *vs*. non-MDs). The statistical significance was set at *p*-value less than 0.05 for the data analysis.

All statistical analyses were performed using EZR (Saitama Medical Center, Jichi Medical University, Saitama, Japan) (12), which is a modified version of R commander, designed to perform statistical functions frequently employed in biostatistics (The R Foundation for Statistical Computing, Vienna, Austria).

## Results

### Participants

**Figure 1:**
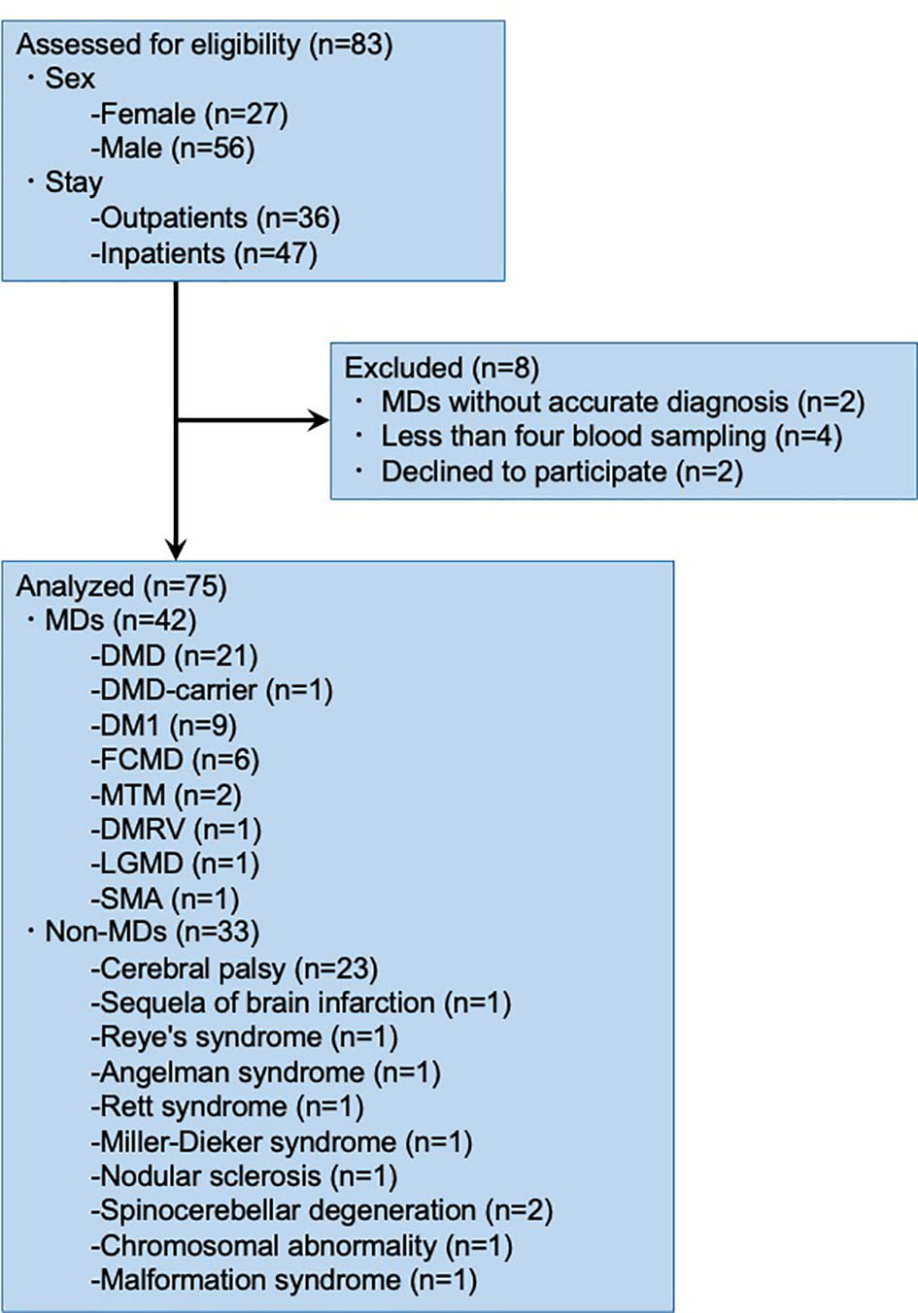
Flow diagram of the study population.

Of the 83 patients, eight were excluded from the study (**Figure 1**). Two patients with MDs were not identified based on the genetic test and muscle biopsy reports; four patients did not undergo blood sampling; and two patients declined to participate in the study. Therefore, we analyzed the data of 75 research participants.

Out of seventy-five patients, twenty-one DMD, one DMD-carrier, nine DM1, six FCMD, two MTM, one DMRV, one LGMD, one SMA, twenty-three cerebral palsy, one sequela of brain infarction, one Reye’s syndrome, one Angelman syndrome, one Rett syndrome, one Miller-Dieker syndrome, one nodular sclerosis, two spinocerebellar degeneration, one chromosomal abnormality, and one malformation syndrome patients were investigated. This study included 24 females (32.0%) and 51 males (68.0%). The median age of the 75 patients was 32, in the range of 12–64, and the mean age was 36.0 years. The proportions of patients with each condition are summarized in **Supplementary Table 1**.

### Anti-SARS-CoV-2 IgG levels with respect to each factor

When analyzed serological responses (anti-SARS-CoV-2 IgG antibodies) between the sexes, no significant differences were observed over time, i.e., on the first day of the second vaccination, and one month, three months, and six months post second vaccination, with *p-*values of 0.98, 0.59, 0.17, and 0.47, respectively (**Table 1**). Similarly, no significant differences were observed in serological responses between inpatients (45 patients, 60.0%) and outpatients (30 patients, 40.0%). In addition, no significant differences were also observed between patients with tracheal positive-pressure ventilation or noninvasive positive-pressure ventilation (27 patients, 36.0%) and those without mechanical ventilation (48 patients, 64.0%), and between tube feeding (30 patients, 40.0%) and oral feeding (45 patients, 60.0%) (**Table 1**).

**Table 1:** Comparative results of anti-SARS-CoV-2 IgG antibody levels in each characteristics.

Characteristic	Number (%)	Anti-SARS-CoV-2 IgG Antibody (BAU/mL) (Median [IQR])							
		Day0	*p-*value	1 month	*p-*value	3 months	*p-*value	6 months	*p-*value
Sex									
Female	24 (32.0%)	233.0 [47.5-331.3]		1556.7 [730.4-3320.9]		422.6 [214.5-811.2]		114.7 [65.7-215.4]	
Male	51 (68.0%)	165.3 [73.9-341.2]	0.984	1297.3 [581.7-2716.8]	0.589	399.8 [176.9-727.6]	0.168	142.2 [64.9-216.4]	0.468
Stay									
Outpatients	30 (40.0%)	221.9 [126.3-454.7]		1701.6 [1059.0-3325.9]		517.9 [264.3-815.3]		142.5 [84.2-208.8]	
Inpatients	45 (60.0%)	128.8 [48.1-313.5]	0.494	846.3 [457.3-2334.1]	0.512	288.2 [142.0-752.9]	0.501	111.4 [52.9-227.3]	0.531
Age (Years)									
<30	35 (46.7%)	250.0 [126.6-499.6]		1767.4 [736.9-4077.2]		570.5 [239.8-1208.5]		142.9 [77.6-263.1]	
>31	40 (53.3%)	126.9 [39.5-229.9]	0.015	980.9 [401.3-2040.8]	0.174	278.4 [144.3-544.0]	0.067	114.5 [52.6-163.1]	0.046
Respiratory status									
NPPV or TPPV	27 (36.0%)	189.8 [76.9-388.0]		846.3 [653.2-1906.1]		348.9 [176.9-524.3]		138.5 [69.8-192.7]	
None	48 (64.0%)	176.3 [65.9-319.9]	0.689	1701.6 [709.4-3492.4]	0.256	470.5 [187.2-817.8]	0.478	129.4 [51.9-231.6]	0.797
Feeding method									
Tube	30 (40.0%)	276.6 [108.3-464.5]		1451.4 [681.8-3102.7]		419.5 [169.7-937.7]		147.7 [69.8-254.2]	
Oral	45 (60.0%)	165.3 [53.4-238.5]	0.061	1346.0 [658.1-2758.3]	0.116	399.8 [202.2-702.3]	0.238	111.4 [59.7-168.4]	0.294
Disease/Disorder									
MDs	42 (56.0%)	163.9 [58.0-333.1]		945.2 [461.7-1780.9]		318.6 [150.9-474.0]		110.7 [55.5-167.1]	
Non-MDs	33 (44.0%)	221.3 [96.9-329.7]	0.28	2138.5 [731.6-4594.1]	0.002	692.3 [237.0-1123.8]	0.008	155.1 [75.4-255.0]	0.221

When we compared the median anti-SARS-CoV-2 IgG between the years of age, a significant difference between those under the age of 30 (250.0 BAU/mL [IQR = 126.6–499.6], 142.9 BAU/mL [IQR = 77.6–263.1]) and those over the age of 31 (126.9 BAU/mL [IQR = 39.5–229.9], 114.5 BAU/mL [IQR = 52.6–163.1]) was observed during the first day of second vaccination followed by six months post vaccination with *p*-values of 0.02 and 0.046, respectively (**Table 1**). No significant differences between patients under the age of 30 and those beyond the age of 31 were observed in one and three months after the second vaccination (*p*-values of 0.17 and 0.07, respectively) (**Table 1**).

### Anti-SARS-CoV-2 IgG levels in MD+/MD-

Later, we evaluated the serological responses of MDs with progressive or degenerative muscular weakness to determine the efficacy and safety of intramuscular mRNA immunization. DMD, DMD-carrier, DM1, FCMD, MTM, DMRV, LGMD, and SMA were among the MDs. Non-MDs mostly consist of SMIDs without progressive or degenerative muscular weakness. Forty-two (56.0%) of the 75 patients had MDs, while 33 (44.0%) did not have MDs. The median age of the patients with and without MDs was 30.5 and 36 years, respectively, and the mean age was 33.8 and 38.7 years, respectively.

**Figure 2:**
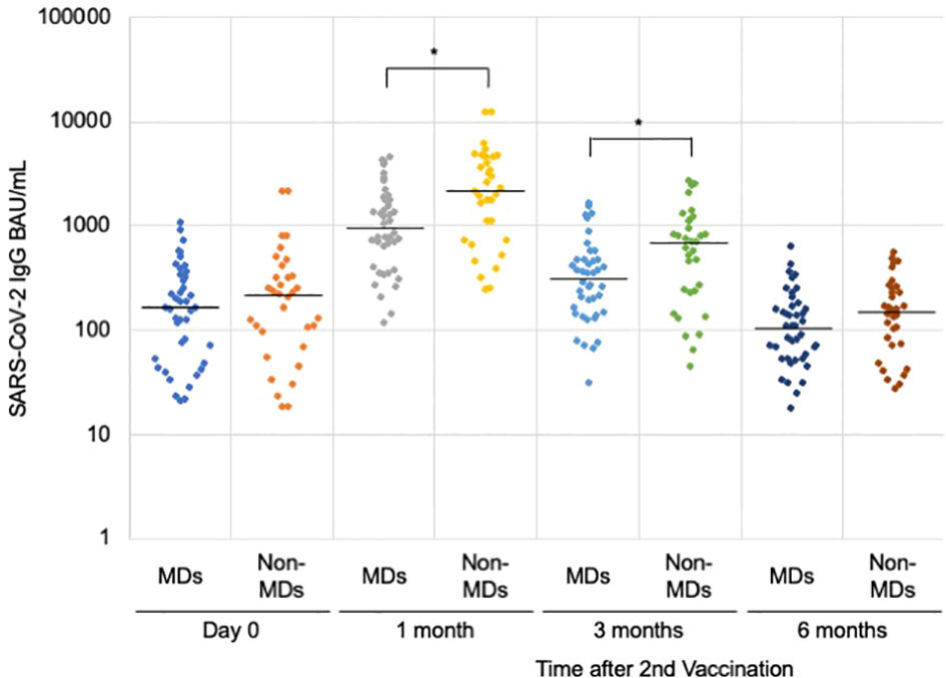
Anti-SARS-CoV-2 IgG antibody levels after the second vaccination. Comparing the muscular disorders (MDs) group (n = 42) with the non-MDs group (n = 33), a significant difference was observed at 1 and 3 months after the second vaccination with the BNT162b2 mRNA vaccine. The solid lines represent the median titers of each group. * indicates *p*-value less than 0.01.

When we compared the median anti-SARS-CoV-2 IgG, those in the MDs group were significantly lower than those in the non-MDs group (945.2 BAU/mL [IQR = 461.7–1,780.9] *vs*. 2,138.5 BAU/mL [IQR = 731.6–4,594.1] in one month, and 318.6 BAU/mL [IQR = 150.9–474.0] *vs*. 692.3 BAU/mL [IQR = 237.0–1,123.8] in 3 months with *p*-values of 0.002, and 0.008, respectively (**Table 1**, **Figure 2**). On the first day and at six months following the second immunization, there was no significant difference between patients with MDs and those without MDs (163.9 BAU/mL [IQR = 58.0–333.1] *vs*. 221.3 BAU/mL [IQR = 96.9–329.7]; and 110.7 BAU/mL [IQR = 55.5–167.1] *vs*. 155.1 BAU/mL [IQR = 75.4–255.0]; *p*-values of 0.28, and 0.22, respectively) (**Table 1**).

### Latency effects for SARS-CoV-2 in MD+/MD-cases

**Figure 3:**
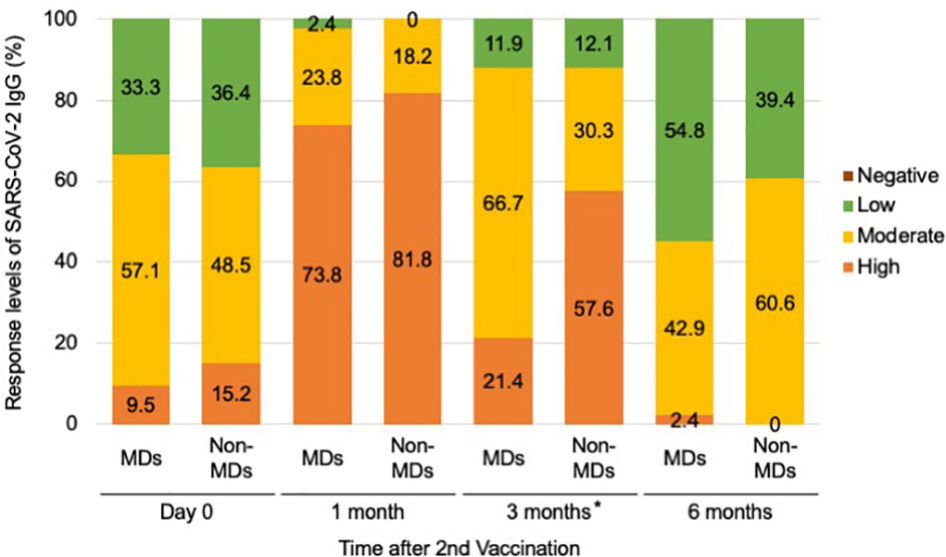
Serological response levels of anti-SARS-CoV-2 IgG antibody after second vaccination in the muscular disorders (MDs) group (n = 42) and non-MDs group (n = 33). Categorical levels of antibody responsiveness based on anti-SARS-CoV-2 IgG antibody quantitative titers that were provided by the manufacturer (Abbott, Chicago, USA) were defined as follows; Negative (<7.1 BAU/mL), Low positive (7.1–119.2 BAU/mL), Moderate positive (119.3–567.9 BAU/mL), and High positive (>568 BAU/mL). Comparing the incidence of immune responses, a significant difference was observed between MD and non-MD groups at 3 months following the second vaccination. * indicates *p*-value less than 0.01.

In addition, when we categorized antibody responsiveness levels depending on the manufacturer guidelines (Abbott, Chicago, USA) to assess latency effects, we observed significant differences between the responsiveness of patients with and without MDs at 3 months following the second vaccination (*p*-values of 0.00253) (**Figure 3**; **Supplementary Figure 1**). Although only nine (21.4%) of 42 patients with MDs demonstrated a strong positive immune response, 19 (57.6%) of 33 patients without MDs demonstrated a high positive immune response during the 3 months after the second vaccination (**Figure 3**). Twenty-eight patients (66.7%) with MDs exhibited moderately favorable immunological responses (**Figure 3**).

### Anti-SARS-CoV-2 IgG levels with respect to each disease

**Figure 4:**
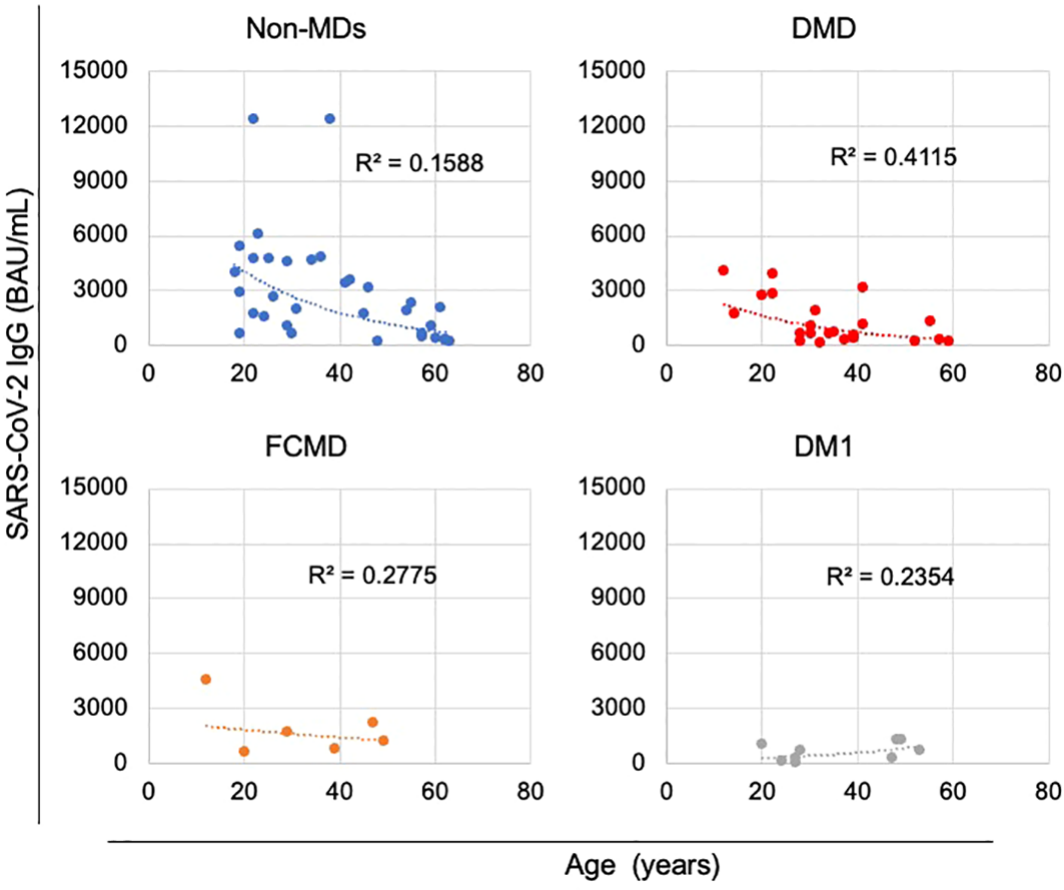
Correlations between anti-SARS-CoV-2 IgG antibody levels and the age of patients during the first month after the second vaccination. Anti-SARS-CoV-2 IgG antibody levels tended to decrease with age in the non-muscular disorder (MD) subgroup (n = 33) and in the DMD subgroup, which included patients with DMD (n = 21) or DMD-carrier (n = 1), unlike that observed in the Fukuyama congenital muscular dystrophy (FCMD) subgroup (n = 6) and the Myotonic dystrophy type 1 (DM1) subgroup (n = 9).

We investigated subgroups within the cohort of MDs to elucidate illness type disparities. When a relationship between subgroups within the MDs cohort and years of age was evaluated, anti-SARS-CoV-2 IgG levels showed declining trend with age in the DMD subgroup, which included patients with DMD and DMD-carrier (**Figure 4**; **Supplementary Figure 2**). The non-MD subgroup and other subgroups, including MTM, DMRV, LGMD, and SMA, exhibited comparable patterns (**Figure 4**; **Supplementary Figures 3, 4**). In contrast, anti-SARS-CoV-2 IgG levels in the FCMD subgroup were unrelated to age at any time following the second dose of vaccination (**Figure 4**; **Supplementary Figure 5**). Additionally, anti-SARS-CoV-2 IgG antibody levels in the DM1 subgroup showed an increasing trend with age (**Figure 4**; **Supplementary Figure 6**).

**Figure 5:**
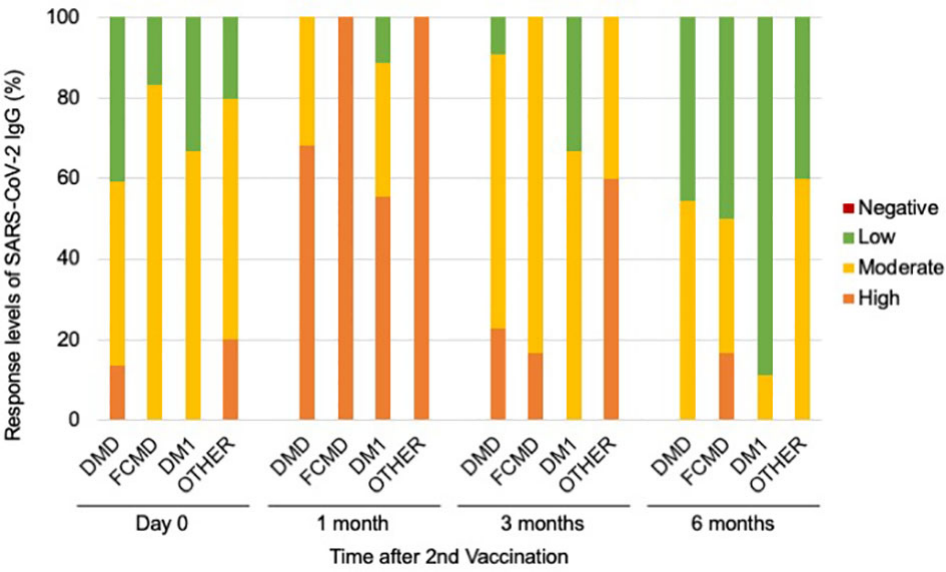
Serological response levels of anti-SARS-CoV-2 IgG antibody after second vaccination in each disease types in MDs. DMD subgroup included DMD and DMD-carrier, and other subgroup including MTM, DMRV, LGMD, and SMA.

When we analyzed the relationship between subgroups within the MDs cohort and immunogenic levels, we found that seven (31.8%) patients with DMD or DMD-carrier and four (44.4%) patients with DM1 had moderate and low immune response during first month after their second dose of vaccination (**Figure 5**). In addition, we observed that 3 months after the second dose of vaccination, 17 (77.3%) DMD or DMD-carrier patients, 5 (83.3%) patients with FCMD, and nine (100%) patients with DM1 had moderate and low immune response levels (**Figure 5**). Clinically significant differences between subgroups within the MDs cohort and in immunogenic levels were difficult to identify because of the small sample size.

### Adverse effects of vaccine against SARS-CoV-2

We retrospectively collected data on systemic and local adverse effects within 7 days of receiving the second mRNA vaccination dosage. We confirmed that 27 patients (36.0%), including 15 patients with MDs, had fever as a systemic adverse effect; 5 (6.7%) patients reported local swelling; 27 (36.0%) patients reported soreness; 7 (9.3%) patients reported redness; and 13 (17.3%) patients reported warmth at or around the injection site. Local adverse effects were noted in three, 22, six, and 10 patients with MDs. No serious or unexpected adverse events were observed in this study.

## Discussion

In this study, we described the immunogenicity of mRNA intramuscular vaccine against SARS-CoV-2 in patients with MDs and non-MDs, including those with SMIDs, to investigate the effect of mRNA intramuscular vaccine on SARS-CoV-2 in patients with MDs who have progressive or degenerative muscular weakness. Despite the fact that patients with MDs had much lower immunological values than patients without MDs, we discovered that many patients with MDs had robust high immune responses during the first month after the second vaccine dose, similar to patients without MDs. In contrast to patients without MDs, the patients with MDs with high immune responses showed a decreased response after 3 months following the second vaccine. Based on these findings, we believe that MDs may be a significant key contributor in predicting the antibody response to SARS-CoV-2 vaccination.

### Contributing factor that can predict the antibody response

The predictive factors for the antibody response to SARS-CoV-2 vaccination remains to be elucidated. Several studies have investigated the causes of weak or high response, namely age, sex, ethnicity, frailty, prior SARS-CoV-2 infection, immunocompromised status, and chronic disease (13–31). The relationship between sex and age for antibody response to SARS-CoV-2 vaccination remains controversial (13–17).

High immune responses were generated in healthcare workers or older residents of a long-term care facility with prior SARS-CoV-2 infection compared to those without prior SARS-CoV-2 infection (18–21). On the other hand, weak immune responses have been generated in immunocompromised patients, including those with autoimmune disease (22, 23), solid organ transplant patients (24, 25), cancer patients treated with programmed cell death protein 1/programmed death-ligand 1 inhibitors or chemotherapy (26), patients with immune-mediated inflammatory disease (27), and patients with chronic lymphocytic leukemia or other hematological malignancies (28). Besides, dialyzed or hemodialyzed patients, frail or disabled nursing home residents also show weak immune responses (29–31). Our study indicated that the sex, place of stay, respiratory status, and feeding method did not affect the antibody response to SARS-CoV-2 vaccination. However, MDs resulted in significant differences of the antibody response to SARS-CoV-2 vaccination. In addition, patients under the age of 30 years had a significantly higher antibody responses than those over the age of 31, on the first day and at six months following the second vaccination. We thought that the significant difference between ages might have occurred due to the percentage of patients with DMD among those with MDs, specifically resulting in progressive muscular degeneration and wasting. Therefore, we speculate that both the quality and quantity of the muscle might affect the immunogenicity of the SARS-CoV-2 vaccination in patients with MDs.

### Longevity of immunogenicity conferred by SARS-CoV-2 vaccination

Dickerson et al. described the course of anti-SARS-CoV-2 IgG antibody levels after the second vaccination in healthcare workers, and a significant decrease in antibody concentration over time was reported (32). Although our study showed a similar decrease in anti-SARS-CoV-2 IgG levels over time, significant differences were observed between the responsiveness of patients with MDs and patients without MDs. In particular, the number of patients with MDs who had high immune responses was reduced by more than that of patients without MDs 3 months after the second vaccination dose. We believe that the immunogenicity of mRNA intramuscular vaccination against SARS-CoV-2 may be difficult to retain for long term in patients with MDs. Taken together, muscular disease may be related to the suppression of the magnitude and latency of the antibody response to SARS-CoV-2 vaccination. In this respect, further research on the molecular mechanism and a longer follow-up period would also be required to determine antibody longevity in patients with MDs.

### Safety of vaccine against SARS-CoV-2

Saita et al. provided real-world evidence on reactogenicity following two doses of BNT162b2 vaccines administered healthcare workers in a large academic hospital in Japan (33). Their results indicated that 42.8% of participants had fever as a systemic adverse effect, 42.9% had local swelling; 87.3% had soreness; 26.5% reported redness; and 37.7% experienced warmth at or around the injection site as local adverse effects. In this study, vaccine-related reactions were partly collected objectively because 27 patients (36.0%) could not answer questions because of intellectual disability. Therefore, we cannot easily compare this with the reactogenicity reported in healthy people. However, we can at least describe that mRNA intramuscular vaccination to SARS-CoV-2 for MDs and non-MDs may be safe because there were no serious adverse effects in our study.

### Limitations in our study

This study has several limitations. First, we cannot compare the immunogenicity with that in healthy people because we did not measure immunological responses in a healthy matched cohort. Takeuchi et al. described immunogenicity of 39 days after the second vaccination in 100 Japanese hospital workers (34). They showed that 100% of workers had values ≥7.1 BAU/mL for anti-SARS-CoV-2 IgG, and the median IQR values of the antibody titers were 1,370.2 BAU/mL [IQR = 733.9–1,866.3]. In this study, 75 (100%) patients had ≥7.1 BAU/mL during the first month after second vaccination dose. In addition, the median IQR values of the antibody titers were 1,346.0 BAU/mL [IQR = 665.8–2,943.9] in all patients. We might be able to state that our patients as a whole showed immunogenicity comparable to that of healthy people. Second, only total IgG antibody levels against the SARS-CoV-2 spike protein S1 subunit were measured in this study. Previous studies have provided not only an assessment of the humoral immune response but also combined analyses of humoral and cellular immunity (35). If our analyses were extended to other antibody subsets (including antibody levels against SARS-CoV-2 nucleocapsid protein, IgM antibody levels against the SARS-CoV-2 spike protein, and neutralizing activity, etc.) or T cell responses to vaccination, we might be able to show other differences. Third, the study size was small. The immunogenicity of intramuscular mRNA vaccination to SARS-CoV-2 in MDs, particularly in subgroups within the MDs, needs to be further investigated in a study with a larger number of cases with MDs. We speculate that disease types in MDs may be important contributing factors in predicting antibody response; therefore, further studies will lead to better management of vaccination in MDs. Fourth, previous studies have also showed the immunogenicity associated with SARS-CoV-2 vaccination in patients with MDs using only an intramuscular mRNA vaccine (7, 8), but not other SARS-CoV-2 vaccines including protein subunit vaccines and viral vector-based vaccines. In addition, we speculate that a 3rd primary dose, with a higher dose, shorter intervals between vaccines such as 3 months, or an altered delivery method, such as nasal spray or aerosol inhalation, might help to improve vaccine responses in patients with MDs. In this respect, SARS-CoV-2 immunization of MD patients requires extensive research, including the various types, doses and intervals of SARS-CoV-2 vaccines.

## Conclusion

We reported the immunogenicity of SARS-CoV-2 mRNA intramuscular vaccination in patients with MDs and non-MDs, including SMIDs. MDs may be a key contributor in predicting the antibody response to SARS-CoV-2 vaccination, but SARS-CoV-2 immunization in patients with MDs warrants further investigation.

## Data availability statement

The raw data supporting the conclusions of this article will be made available by the authors, without undue reservation.

## Ethics statement

The studies involving human participants were reviewed and approved by Nagara Medical Center. Written informed consent to participate in this study was provided by the participants’ legal guardian/next of kin.

## Author contributions

RK analyzed subjects and drafted the manuscript. MF contributed to design the study, analyze subjects, and draft the manuscript. KM, KY, HM, and KT contributed to design the study, analyze subjects, and review and edit the manuscript. JI contributed to analyze subjects. All authors contributed to the article and approved the submitted version.
