# Supplementary figures and images for: Immunogenicity of SARS-CoV-2 mRNA intramuscular vaccination in patients with muscular disorders
Source: Front Immunol. 2023 Feb 7;14:1103196. doi: 10.3389/fimmu.2023.1103196 (PMC9941142; doi:10.3389/fimmu.2023.1103196)

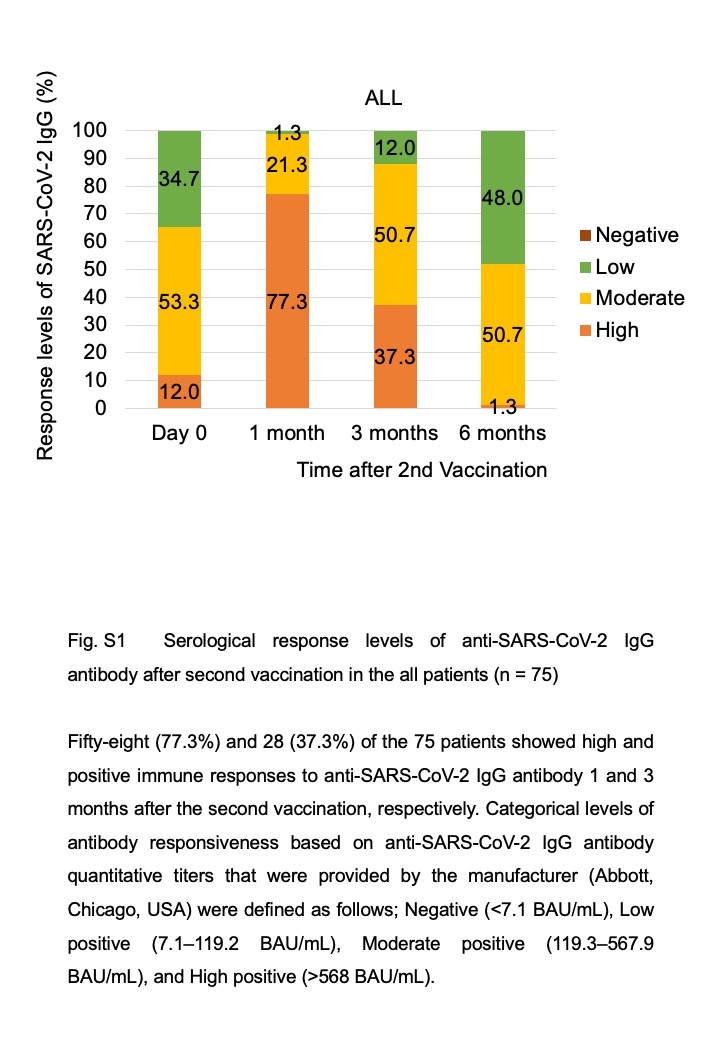

Supplement: Supplementary file 1 [file Image_1.jpeg]

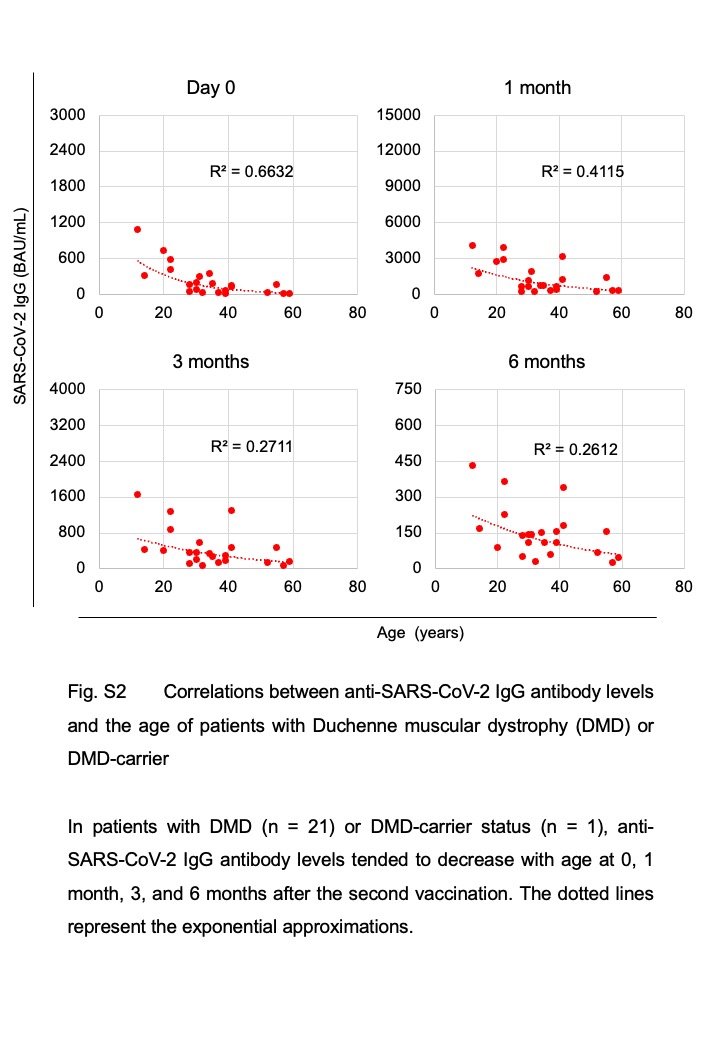

Supplement: Supplementary file 2 [file Image_2.jpeg]

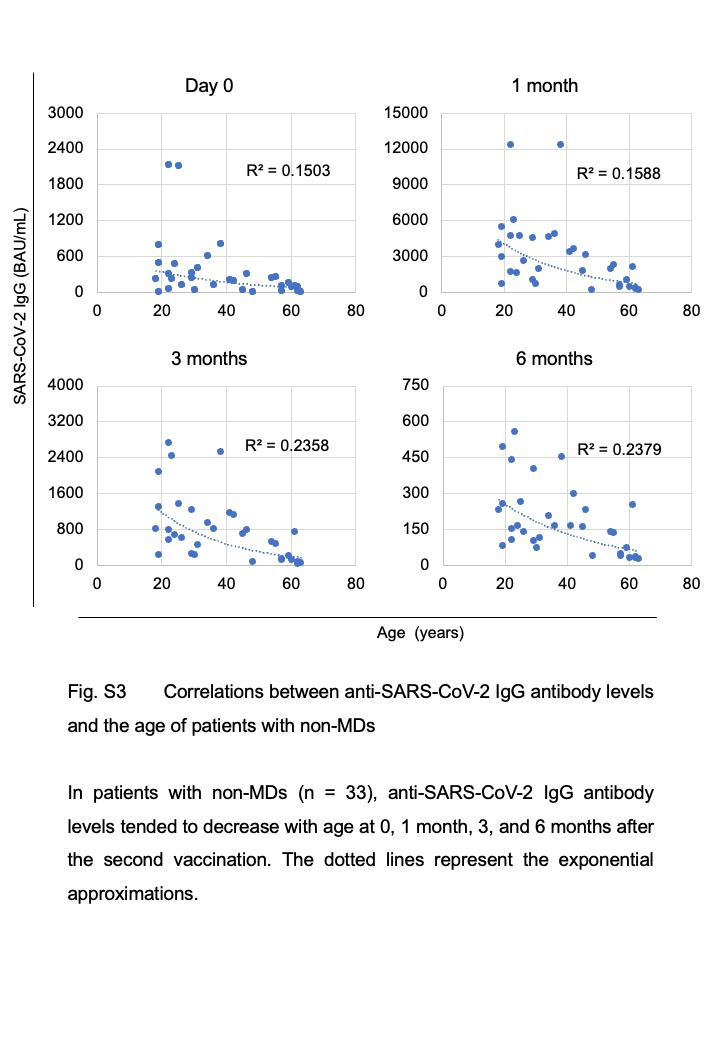

Supplement: Supplementary file 3 [file Image_3.jpeg]

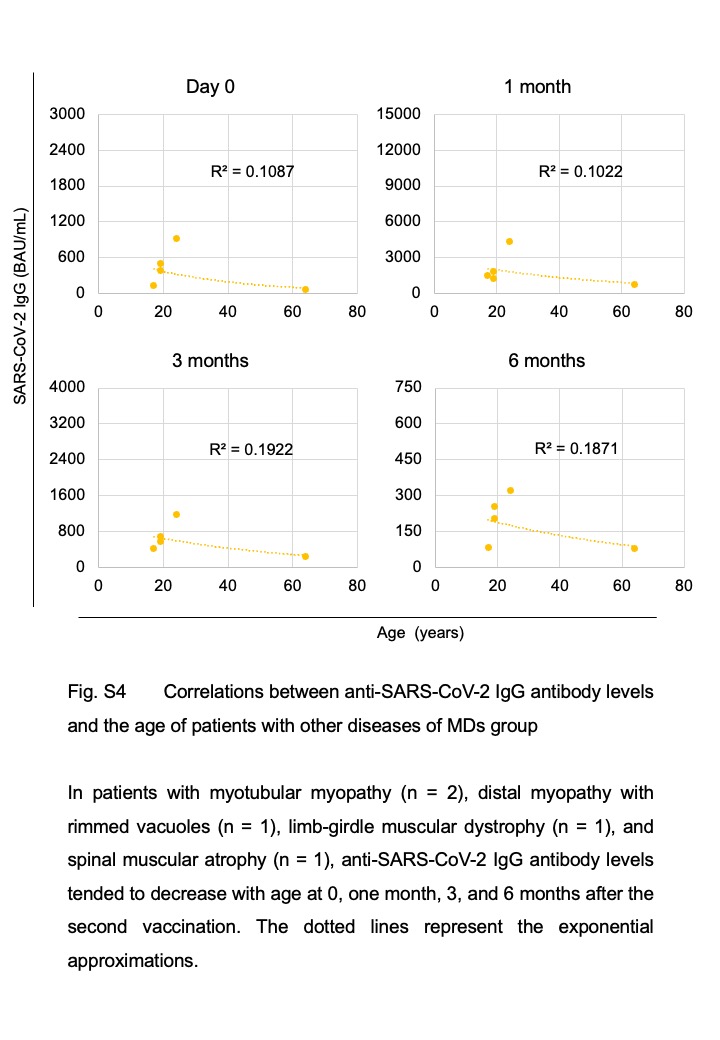

Supplement: Supplementary file 4 [file Image_4.jpeg]

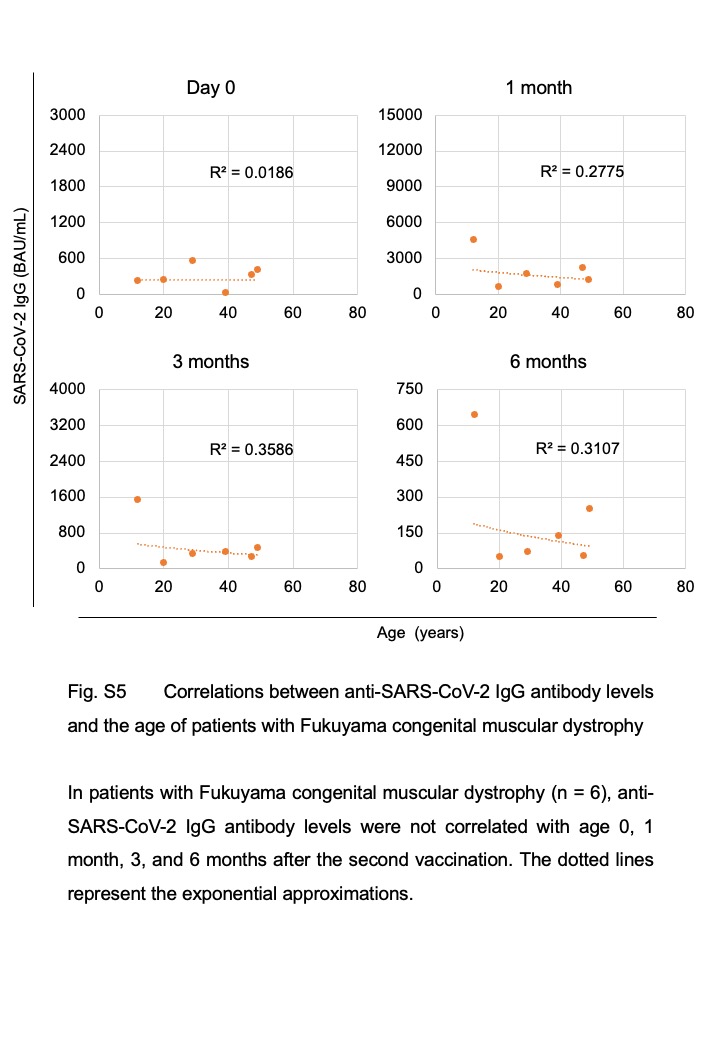

Supplement: Supplementary file 5 [file Image_5.jpeg]

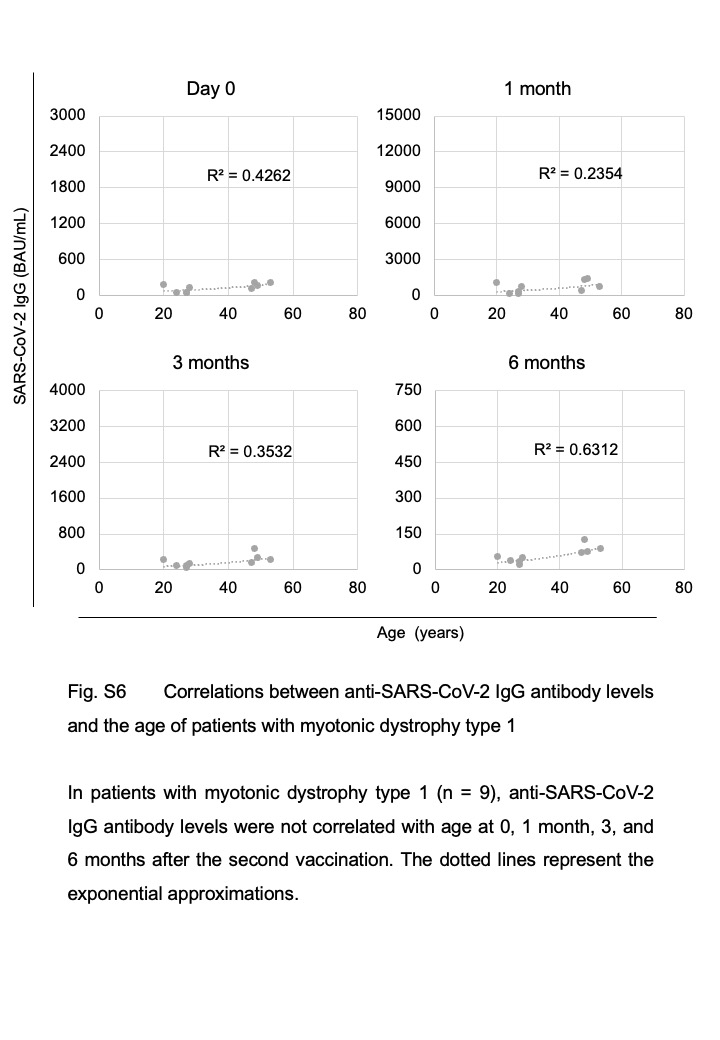

Supplement: Supplementary file 6 [file Image_6.jpeg]
